# Effects of CPAP on right ventricular myocardial performance index in obstructive sleep apnea patients without hypertension

**DOI:** 10.1186/1465-9921-7-22

**Published:** 2006-02-06

**Authors:** Nese Dursunoglu, Dursun Dursunoglu, Sibel Özkurt, Sükrü Gür, Güllü Özalp, Fatma Evyapan

**Affiliations:** 1 Goteborg University Sahlgrenska Hospital Sleep Laboratory, Göteborg, Sweden; 2 Pamukkale University Medical Faculty, Department of Chest, Denizli, Turkey; 3 Pamukkale University Medical Faculty, Department of Cardiology, Denizli, Turkey

## Abstract

**Objectives:**

Obstructive sleep apnoea (OSA) might cause right ventricular dysfunction and pulmonary hypertension. We aimed to determine the effects of nasal continuous positive airway pressure (CPAP) therapy on right ventricular myocardial performance index (MPI) in OSA patients without hypertension.

**Methods:**

49 subjects without hypertension, diabetes mellitus, any cardiac and pulmonary disease had overnight polysomnography and echocardiography. In 18 moderate-severe OSA (apnea-hypopnea index ≥ 15) patients, right ventricular free wall diameter (RVFWD) was measured by M-mode, and right ventricular MPI was calculated as (isovolumic contraction time+ isovolumic relaxation time) / pulmonary ejection time using Doppler at baseline and after 6 months CPAP therapy.

**Results:**

Mean age was 46.5 ± 4.9 year. Patients had high body mass index (BMI: 30.6 ± 4,0 kg/m^2^), but there was no change in either BMI or blood pressures after 6 months. Right ventricular end-diastolic and end-systolic diameters were in normal limits at baseline, and did not change after CPAP usage. Baseline RVFWD (7.1 ± 2.1 mm) significantly decreased after CPAP therapy (6.2 ± 1.7 mm, p < 0.001). 15 of patients (83%) had right ventricular diastolic dysfunction at baseline, and it was completely improved in 11 of them (73%) by CPAP usage. Right ventricular global dysfunction was shown in 11 patients (61%) with a high MPI (62.2 ± 9.3%) at baseline; and MPI was significantly decreased after CPAP therapy (47.3 ± 8.4%, p < 0.0001), and it was completely corrected in 4 of them (36%).

**Conclusion:**

CPAP therapy significantly decreases RVFWD and improves right ventricular diastolic and global functions (MPI) in OSA patients without hypertension.

## Introduction

The obstructive apneic event is associated with considerable breathing efforts against totally or partially occluded upper airway. Obstructive sleep apnoea (OSA) might lead to cardiovascular complications such as heart failure, left/right ventricular dysfunction, myocardial infarction, arrhythmias, systemic and pulmonary hypertension, that all increase morbidity and mortality [1-10]. Early recognition and appropriate therapy of ventricular dysfunction is advisable to prevent further progression to heart failure and death [11,12].

During an obstructive apnea, large negative intrathoracic pressures are generated during inspiratory efforts, which increase transmural pressures across the myocardium, thus increasing afterload. An increase in preload and pulmonary congestion may also occur due to increased venous return. The presence of hypoxemia decreases oxygen delivery to the myocardium, which may promote angina and arrhythmias. Also, frequent arousals from sleep lead to increase sympathetic activity. Other responsible mechanisms include impaired vagal activity, increased platelet aggregability, insulin resistance, and endothelial dysfunction with reduced endogenous nitric oxide production [13]. Continuous positive airway pressure (CPAP) is known to maintain upperairway patency during sleep by increasing transmural pressure of upper airways, and treatment of OSA by CPAP improves cardiac function and quality of life [14-17].

Ventricular dysfunction could not be detected clinically, but could just be detected echocardiographically. Myocardial performance index (MPI) reflects both systolic and diastolic functions (global function) of the ventricle [18,19]. In the presence of ventricular global dysfunction, MPI increases in contrast to ejection fraction (EF) of the ventricle. MPI is reproducible and a simple non-invasive method for the estimation of ventricular global function.

In the present study, we aimed to determine the effects of nasal CPAP after 6 months of therapy on right ventricular MPI in moderate-severe OSA patients without hypertension.

## Methods

### a) Study population

49 subjects admitted to sleep clinic with symptoms of nocturnal snoring and/or excessive day-time sleepiness taken into the study. A detailed sleep and cardiovascular anamnesis of the patients were recorded. Sleep cycle, nutritional status, medications, alcohol usage and family anamnesis were also questioned. Epworth sleepiness scale (ESS) [20] was questioned to all patients, and patients having high scores (ESS ≥ 10) were taken into the sleep study.

Physical examination was performed to all subjects at baseline and at 6 months. Systolic (SBP) and diastolic (DBP) blood pressures were measured in the sitting position on the right arm using a sphygmomanometer (Erka, Germany), after at least 5 min of rest. Hypertension was identified as blood pressure ≥ 140/90 mmHg or being used antihypertensive drugs. Heart rate per minute (HR) was measured in the sitting position, and body mass index (BMI) of the patients were calculated as weight divided to height square (kg/m^2^).

Pulmonary function tests (Sensor medics 2400, Netherlands), arterial blood gas analysis (ABL 30, Kopenhagen) were performed to all patients at rest. A 12-leadsurface ECG was taken from every subject, and all of them were in sinus rhythm. Also, all of subjects underwent treadmill exercise test and, the test was normal for each subject.

Symptoms of nocturnal snoring and/or excessive day-time sleepiness were accepted as inclusion criteria. Exclusion criteria of the study were the presence of 1- any known cardiac and lung disease, 2- hypertension, 3- diabetes mellitus, 4- angina pectoris, 5- atrial fibrillation or any arrhythmias, 6- chronic renal and hepatic diseases, 7- serum electrolytes imbalances.

### b) Polysomnography (PSG)

All patients underwent an entire night of diagnostic PSG while breathing room air, followed a second night of PSG with nasal CPAP titration [21]. The portable, limited sleep study performed with the Emblettadevice [22], consisted of the following: 1) nasal pressure detectorusing nasal cannulae/pressure transducer system, recording thesquare root of pressure as an index of flow; 2) thoraco-abdominalmovement detection through two piezoelectric belts; 3) fingerpulse oximeter; and 4) body position detection.

Apnea was defined as total obstruction of oronasal airflow ≥ 10 seconds, an hypopnea was defined as decrease of airflow at least 50%, and desaturation was accepted as a decrease of ≥ 4% in oxygen saturation [23]. Desaturation index (DI) was identified as number of oxygen desaturation events per hour of sleep. In this study, 21 non-hypertensive subjects with AHI ≥ 15 were diagnosed as moderate-severe OSA [24].

### c) Nasal continuous positive airway pressure (CPAP)

Patients with severe OSA were prescribed nasal CPAP (Remstarplus, Respironics Corporation; Murrysville, Pennsylvania, USA), including use of a heated humidifier. The CPAP machines were equipped with compliance monitors that measured CPAP use, in order to observe CPAP usage/compliance at home. Routine troubleshooting took place in an effort to maximize compliance with nasal CPAP. Patients were considered to be CPAP compliant if they used CPAP an average ≥ 3.5 h per night at the 6-month follow-up.

### d) Echocardiographic measurements

Echocardiograms were obtained following the diagnosis of OSA and prior to initiation of CPAP, and at 6 months. All measurements were performed with the subjects in the left lateral decubitus position by M mode, two dimensional (2D), and Doppler ultrasound echocardiography in the morning. The ultrasound equipment used was Contron Sigma Iris with a 2.5-MHz probe. The duration of the examination was at least 20 minutes. The ventricular diameters, volumes and functions were measured according to the recommendations of the American Society of Echocardiography [25]. Basic measurements of left/right ventricular dimensions in diastole and systole, thickness of interventricular septum (IVS), left ventricular posterior wall (LVPW) and right ventricular free wall diameter (RVFWD) were measured by M-mode technique.

Early (E) and atrial (A) trans-mitral/tricuspid maximal flow velocities, the ratio (E/A) and deceleration time (DT) of E was registered. Isovolumetric relaxation time (IVRT) was measured by the continuous wave Doppler technique. The velocity of mitral/tricuspid flow propagation (VPR) was estimated using color Doppler M-mode. The left/right ventricular MPI was calculated as (isovolumic contraction time of left/right ventricle + IVRT of left/right ventricle) / (aortic/pulmonary ejection time) by Doppler.

### e) Statistical analysis

Measurements are expressed as mean ± SD. A Wilcoxon test for related measurements was used to compare primary outcome variables of both IVS, LVPW, RVFWD and ventricular functions (LVEF, MPI) at baseline and 6 months. Also, Mc Nemar chi-square test was used for comparison of number of obstructive sleep apnea patients without hypertension according to right ventricular hypertrophy and diastolic or global dysfunctions at baseline (before treatment) and after 6 months of CPAP usage. A p value of < 0.05 was regarded as significant.

## Results

18 of 21 patients were compliant with CPAP therapy, which we defined as ≥ 3.5 h of nightly use. At the end of the study the mean daily CPAP usage of 18 patients, was 5.6 ± 1.7 h per night. The 3 patients who were non-compliant with nasal CPAP were excluded. So, 14 males (78%) and 4 females (22%) with moderate-severe OSA without hypertension were included in the study. Mean age of patients was 46.5 ± 4.9 year. None of them were using alcohol, and 55% of them were smoking cigarettes. Baseline characteristics of OSA patients were shown in table 1. Study patients had high BMI (mean 30.6 ± 4,0 kg/m^2^), however there were no change in either BMI or blood pressures (BP) after 6 months (see table 2). Mean AHI was 50.1 ± 11.6 per h, average O_2 _saturation was 80.1 ± 2.6% and sleep duration with oxygen saturation <90% was 66.4% in the patients (table 1).

**Table 1:** Baseline characteristics of obstructive sleep apnea patients without hypertension at pre-treatment of continuous positive airway pressure (CPAP).

**Variables**	**Data**
Subjects n	18
Mean age yrs	46.5 ± 4.9
Male n, (%)	14 (78)
Female n, (%)	4 (22)
BMI kg/m^2^	30.6 ± 4,0
SBP mmHg	120.3 ± 10.1
DBP mmHg	82.0 ± 4.1
HR pulse*min^-1^	82.4 ± 13.4
AHI per h	50.1 ± 11.6
DI per h	37.8 ± 18.0
MinSatO_2 _(%)	73.8 ± 18.9
AverageSatO_2 _%	80.1 ± 2.6
*Sleep duration %	66.4
CPAP usage h/night	5.6 ± 1.7
CPAP pressure cmH_2_O	11.3 ± 2.5

Data are presented as n, n(%) or mean ± SD. BMI: body mass index, SBP: systolic blood pressure, DBP: diastolic blood pressure, HR: heart rate, AHI: apnea hypopnea index, DI: desaturation index, SatO_2_: saturation of nocturnal arterial oxygen, NS: nonsignificant. *SatO_2 _< 90%

**Table 2:** Basic characteristics and left ventricular structure and function indices in overall obstructive sleep apnoea patients before and after 6 months of continuous positive airway pressure (CPAP) treatment.

	**CPAP treatment**		**p-value**
**Overall (n = 18)**	**Before**	**After**	
**Basic characteristics**			
BMI kg*m^-2 ^(<30)*	30.6 ± 4,0	31,0 ± 3,5	NS
SBP mmHg (<140)*	120.3 ± 10.1	119,1 ± 10,2	NS
DBP mmHg (<90)*	82.0 ± 4.1	81.2 ± 3,8	NS
**Left ventricular structure**			
Left atrium mm (19–40)*	35.1 ± 3.2	36.0 ± 3.0	NS
IVS thickness mm (6–11)*	10.3 ± 1.1	10,4 ± 1,0	NS
LVPW thickness mm (6–11)*	10.1 ± 0.8	10,0 ± 0,9	NS
LVEDD mm (37–56)*	47.1 ± 4.1	46.1 ± 4.3	NS
LVESD mm (19–40)*	32.2 ± 4.8	31.8 ± 4.7	NS
**Diastolic functions**			
E/A ratio (>1)*	1.6 ± 0.1	1.6 ± 0.2	NS
IVRT ms (<100)*	80.0 ± 12.0	81.1 ± 12.6	NS
DT ms (<220)*	180.2 ± 16.5	179.5 ± 16.8	NS
VPR cm*s^-1 ^(>55)*	89.2 ± 10.6	88.6 ± 10.3	NS
**Systolic function**			
LVEF % (55–75)*	64.0 ± 4.9	64,1 ± 4,7	NS
**Global function**			
MPI % (39 ± 5)*	37.2 ± 6.0	36.8 ± 5.8	NS

Data are presented as mean ± SD. *Normal values, BMI: Body mass index, SBP: Systolic blood pressure, DBP: Diastolic blood pressure, IVS: Ýnterventricular septum, LVPW: Left ventricular posterior wall, LVEDD: Left ventricular end diastolic diameter, LVESD: Left ventricular end systolic diameter, E/A: Ratio of early and lately mitral flow velocity, IVRT: Ýsovolumic relaxation time, DT: Mitral deceleration time, VPR: Velocity of mitral flow propagation, LVEF: Left ventricular ejection fraction, MPI: Myocardial performance index of left ventricle, NS: nonsignificant.

### Compliance of CPAP

The major reasons for noncompliance were mask discomfort or pressure intolerance. All subjects were offered heated humidity, and efforts were made in all subjects to improve comfort and compliance with therapy. Average CPAP pressure after 6 months of therapy did not change from the initial CPAP pressure that was prescribed (11.3 ± 2.5 cmH_2_O and 11.0 ± 2,3 cmH_2_O).

### Echocardiographic findings before and after CPAP therapy

Baseline characteristics and left ventricular structure and function indices in OSA patients before and after 6 months of CPAP treatment were shown in table 2. All echocardiographic left ventricular structural (e.g. IVS, LVPW diameters) and functional (e.g. E/A ratio, LVEF and MPI) parameters were in normal limits, and all of them did not significantly change after 6 months of CPAP therapy in OSA patients without hypertension.

Right ventricular structure and function indices in the patients before and after 6 months of CPAP treatment were shown in table 3. Although right atrial and right ventricular end-diastolic diameters were in normal limits, and both of them did not significantly change; right ventricular free wall diameter at baseline (7.1 ± 2.1 mm) significantly decreased (6.2 ± 1.7 mm, p < 0.001) after CPAP usage.

**Table 3:** Right ventricular structure and function indices in overall obstructive sleep apnoea patients before and after 6 months of continuous positive airway pressure (CPAP) treatment.

	**CPAP treatment**		**p-value**
**Overall (n = 18)**	**Before**	**After**	
Right atrium mm	23.9 ± 3.0	23.7 ± 3.2	NS
RVFWD mm (5–8)	7.1 ± 2.1	6.2 ± 1.7	0.001
RVEDD mm (9–26)	25.4 ± 3.6	25.0 ± 3.3	NS
E-velocity (m/s)	0.4 ± 0.1	0.6 ± 0.1	0.0001
A-velocity (m/s)	0.6 ± 0.13	0.5 ± 0.9	0.001
E/A ratio (>1)*	0,7 ± 0.1	1.2 ± 0.2	0.0001
IVRT ms (<100)*	106.9 ± 24.0	91.1 ± 18.6	0.001
DT ms (<220)*	245.5 ± 61.0	210.2 ± 46.8	0.001
VPR cm*s^-1 ^(>55)*	41.1 ± 9.9	60.9 ± 10.2	0.0001
MPI % (39 ± 5)*	62.2 ± 9.3	47.3 ± 8.4	0,0001

Data are presented as mean ± SD. *Normal values, RVFWD: Right ventricular free wall diameter, RVEDD: Right ventricle end-diastolic diameter, E- velocity: Early tricuspid flow velocity, A- velocity: Lately tricuspid flow velocity, E/A: Ratio of early and lately tricuspid flow velocity, IVRT: Ýsovolumic relaxation time, DT: tricuspid deceleration time, VPR: Velocity of tricuspid flow propagation, MPI: Myocardial performance index of right ventricle, NS: nonsignificant.

Although patients had right ventricular diastolic dysfunction with an increased mean value of diastolic function parameters such as E/A ratio, IVRT, DT and VPR at baseline; average value of all those parameters significantly decreased by CPAP therapy. Also, right ventricular MPI (62.2 ± 9.3%) was significantly decreased after 6 months of CPAP usage (47.3 ± 8.4%, p < 0.0001), but it did not come to the normal limits, and it still reflected right ventricular global dysfunction (see table 3).

Comparison of number of OSA patients without hypertension according to right ventricular hypertrophy (RVH) and diastolic or global dysfunctions at baseline (before treatment) and after 6 months of CPAP usage was shown in table 4. Three of 18 patients (17%) had RVH, and although RVH had improved by CPAP usage, there was still 3 patients having RVH at the end of 6 months. Fifteen of 18 patients (83%) had right ventricular diastolic dysfunction, and 11 of them (73%) completely improved by CPAP usage. Right ventricular global function (MPI) was shown in 11 patients (61%), and also 4 of them (36%) completely improved by CPAP therapy.

**Table 4:** Comparison of number of obstructive sleep apnea patients without hypertension according to right ventricular hypertrophy (RVH) and diastolic or global dysfunctions at baseline (before treatment) and after 6 months of continuous positive airway pressure (CPAP) usage.

	**CPAP treatment**		**p- value**
**Overall (n = 18)**	**Before**	**After**	
Patients with RVH n (%)	3 (17)	3 (17)	NS
Diastolic dysfunction n (%)	15 (83)	4 (22)	0,0001
Global dysfuntion n (%)	11 (61)	7 (39)	0,001

Data are presented as n or n (%), NS: nonsignificant.

## Discussion

In our previous studies, we showed right and left ventricular global dysfunctions in OSA patients with an increased MPI [3,4]. Arterial hypertension, obesity, diabetes mellitus and coronary artery disease which often have coexistince with OSA are independent predictors of ventricular dysfunction. On the other hand, it's well known that OSA might cause cardiovascular complications such as heart failure, myocardial infarction, arryhthmias, systemic and pulmonary hypertension which all increase mortality and morbidity [1-10]. Continuous positive airway pressure is known to maintain upperairway patency during sleep by increasing transmural pressure of upper airways, and treatment of OSA by CPAP improves cardiac function and quality of life [14-17]. In the present study, we aimed to determine the effects of nasal CPAP after 6 months of therapy on right ventricular structure and global function (MPI) in moderate-severe OSA patients without hypertension.

In our study, we excluded systemic hypertension, diabetes mellitus, coronary artery disease and any pulmonary diseases including chronic obstructive pulmonary disease, and there were no significant differences in BMI or BPs after 6 months of therapy. Thus, we assessed the ventricular structure and functions (MPI) in uncomplicated (isolated) OSA patients.

Left ventricular systolic and/or diastolic functions provide prognostic information in the patients. In our study, left ventricular diameters and both systolic and diastolic functions of all subjects were in normal limits and did not significantly change after CPAP therapy. However, right ventricular free wall diameter significantly decreased after CPAP usage. Eleven of 15 patients having diastolic dysfunction (73%) and 4 of 11 patients having right ventricular global dysfunction (36%) completely improved by CPAP therapy.

The relation of OSA to right heart structure and function is controversial. The prevalence of RVH by echocardiography in sleep apnea was ranged from 0 to 71% [26]. In the present study, 3 of 18 patients (17%) had RVH, and it was significantly improved by CPAP usage inspite of any change in the number of patients having RVH. It has been argued that concomitantchronic pulmonary disorders are required for sleep apnea to causeright heart failure [27-31]. However, Sannerand colleagues demonstrated that sleep apnea was independentlyassociated with depressed right ventricular ejection fraction by radionuclideventriculography after adjusting for lung function, age, bodymass index, sex, blood gas analysis, pulmonary artery pressure, and left ventricular ejection fraction [32]. Hanly and colleagues found no difference in right or left ventricular dimensions betweennonapneic snorers and subjects with OSA [33].

The reasons for the disparate conclusions of the prior studies examining RVH, systolic function, and right ventricular enlargementare not certain. In a study, right atrial and ventricular dimensions, and right ventricular systolicfunction were not found to be significantly different betweensubjects with sleep-disordered breathing and the low respiratory disturbance index subjects, but this study indicated that sleep-disordered breathing was associated with increased right ventricular wall thickness in a general population [34]. Right atrial and ventricular diameters of our patients without hypertension were in normal limits at baseline, and none of them significantly have changed by CPAP usage.

In many studies beneficial effect of CPAP on cardiac functions have been shown [35-37]. These may include several factors, such as improved myocardialoxygen delivery, decreased sympathetic activity, left ventriculartransmural pressure, and afterload. In a study, Arabi et al. had proved that systemic hypertension developed in hypoxic situation in normotensive cases, and furthermore they showed a decrease in the adrenergic mediators in patients having CPAP therapy for OSA [38]. Also, Cloward TV et all. showed a regression of left ventricular hypertrophy by 6 months of CPAP therapy, but not in the left and right atrial enlargement [39]. Recent placebo-controlled trials have revealed reductions of up to 10 mmHg in systolic and diastolic pressures with CPAP therapy [40-42]. However, we did not show any decrease in BPs with CPAP therapy in our non-hypertensive OSA patients. The reason of no change in BP might be due to the small sample size of our study population, and it might depend on totally normotensive patients. In our another study, we showed that CPAP therapy in OSA patients with hypertension did not decrease BPs and heart rates acutely, but reduced the variability of these parameters during sleep [17].

Doherty LS, et all. performed a long-term follow-up study of 168 patients with OSA who had begun receiving CPAP therapy at least 5 years previously, most of whom had been prospectively followed up, having been the subject of an earlier report on cardiovascular risk factors in OSA patients with the average follow-up period 7.5 years [43]. They compared the cardiovascular outcomes of those patients who were intolerant of CPAP (untreated group, 61 patients) with those continuing CPAP therapy (107 patients). Deaths from cardiovascular disease were more common in the untreated group than in the CPAP-treated group during follow-up (14.8% vs 1.9%, respectively; p < 0.009), but no significant differences were found in the development of new cases of hypertension, cardiac disorder, or stroke. Total cardiovascular events (ie, death and new cardiovascular disease combined) were more common in the untreated group than in the CPAP-treated group (31% vs 18%, respectively; p < 0.05). They concluded that their results support a protective effect of CPAP therapy against death from cardiovascular disease in patients with OSA.

Our study does not clarify the reasons of right ventricular diastolic and global dysfunctions in OSA patients without hypertension. Right ventricular diastolic filling is a complex event that is influenced by several factors, such as right ventricular relaxation, right ventricular compliance, right atrium contraction force and pulmonary artery resistance. Thus, right ventricular dysfunction might be the result of a variety of these impairments. In our study patients, intermittent nocturnal hypoxemia might have caused RVH and/or right ventricular dysfunction, since they had more hypoxic duration in their sleep with the highest percent of sleep duration below 90% of oxygen saturation.

Since the systolic and diastolic dysfunction frequently coexist, it was shown that a combined measure of ventricular performance with calculation of MPI might be more reflective of overall cardiac dysfunction than systolic or diastolic measures alone. MPI is a simple and reproducible non-invasive method for diagnosis of ventricular global dysfunction in patients with OSA.

In the present study, a very noticeable finding is the improvement of right ventricular free wall dimension and global dysfunction with a significantly decreased MPI by CPAP therapy, even if 6 months usage.

## Conclusion

OSA patients, especially having diastolic dysfunction, is in an increased risk of heart failure, since the systolic and diastolic dysfunction frequently coexist. A combined measure of ventricular performance with the calculation of MPI might be more reflective of overall cardiac dysfunction than systolic or diastolic measures alone. CPAP therapy significantly decreases right ventricular free wall thickness and improves global dysfunction with a significantly decreased MPI even if 6 months of CPAP usage. We consider that much longer-term usage of CPAP therapy may achieve complete improvement of right ventricular global dysfunction in patients with OSA.

### Study limitations

Small number of patients in our study population is an important study limitation to determine the effects of CPAP on RVH and right ventricular dysfunction. Also, our sleep clinic population may not reflect the findings in the general community. A larger population of study subjects would be helpful to determine the effects of CPAP on right ventricular structure and function.

**Figure 1:**
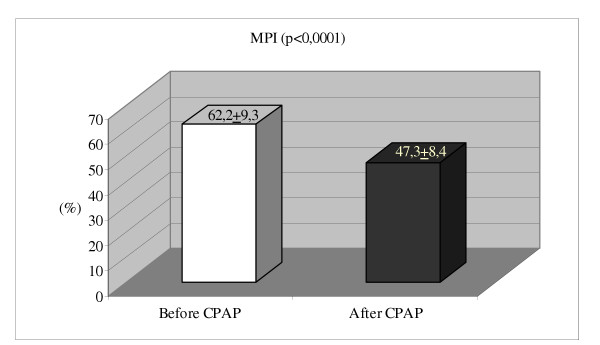
Comparison of right ventricular myocardial performance index (MPI) before and after 6 months CPAP therapy in obstructive sleep apnea patients without hypertension.

**Figure 2:**
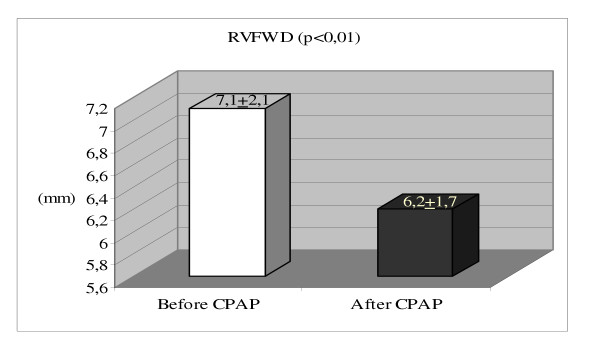
Comparison of right ventricular free wall dimension (RVWFD) before and after 6 months CPAP* therapy in obstructive sleep apnea patients without hypertension.
